# Improving Usability of the Pediatric Code Cart by Combining Lean and Human Factors Principles

**DOI:** 10.1097/pq9.0000000000000676

**Published:** 2023-08-07

**Authors:** Maria Frazier, Kristen Webster, Maya Dewan, Tamara Hutson, Kelly Collins, Tina Fettig, Taylor Grooms, Mary Cordray, Ken Tegtmeyer

**Affiliations:** From the * Department of Pediatrics, Division of Critical Care Medicine, Cincinnati Children’s Hospital Medical Center, Cincinnati, Ohio; † Patient Safety, Regulatory, and Accreditation, James M. Anderson Center for Health Systems Excellence, Cincinnati Children’s Hospital Medical Center, Cincinnati, Ohio; ‡ Department of Pediatrics, University of Cincinnati College of Medicine, Cincinnati, Ohio; § James M. Anderson Center for Health Systems Excellence, Cincinnati Children’s Hospital Medical Center, Cincinnati, Ohio; ¶ Division of Pharmacy, Cincinnati Children’s Hospital Medical Center, Cincinnati, Ohio; ‖ Department of Pediatrics, Division of Cardiology, Cincinnati Children's Hospital Medical Center, Cincinnati, Ohio.

## Abstract

Cardiac arrests are common in hospitalized children. Well-organized code carts are needed during these events to help staff efficiently find supplies and medications for the patient. This study aimed to improve the efficiency and utilization of the code cart at a major academic pediatric medical center.

**Methods:**

This quality improvement project used a phased approach to redesign the code cart. A multidisciplinary team used Lean and Human Factors principles to improve the efficiency and intuitiveness of the redesigned cart. Nurses and respiratory therapists participated in simulations asking for certain supplies with the original and redesigned code cart and filled out surveys for feedback on each code cart. Facilitators measured retrieval times during each simulation.

**Results:**

We performed 10 simulations with the original code cart and 13 with the redesigned code cart. Staff could find intraosseous access equipment more quickly (23.9 versus 46.4 seconds; *P* = 0.003). In addition, staff reported they were less likely to open the wrong drawer or grab the wrong equipment and that the redesigned code cart was overall more well organized than the original code cart. Finally, the redesigned code cart reduced the cost by over 800 dollars per full cart restock.

**Conclusion:**

Revising the code cart using Lean and Human Factors improves efficiency and usability and can contribute to cost savings.

## INTRODUCTION

Each day there are more than 40 cardiac arrests in hospitalized children in the United States.^1^ In-hospital cardiac arrest requires rapid and efficient coordination of personnel and equipment to assure optimal performance and outcomes. Code carts (also known as crash carts or resuscitation carts) are transported to the patient’s side per life support protocols and are vital resources in the hospital setting. Code carts commonly consist of drawers on wheels containing emergency medication and equipment. Most facilities organize code carts according to medical interventions, such as intubation, intravenous access, and medications.^2,3^ While serviceable, our institution’s original code cart design lacked intuitive design input from clinical staff members. This fact and the standardization of materials and supplies in the code cart were barriers that limited the usability and efficiency of the cart in our institution and others.^2,4,5^ These delays can lead to obstructions to providing lifesaving care, patients receiving expired medications, and worse outcomes.^4,5^ In response to these barriers, there are ongoing efforts by health care providers to standardize the code cart.^6–8^

As hospitals strive to improve patient safety and outcomes, efforts to increase health care value by simultaneously decreasing cost and improving the quality of care are important components.^9–12^ Lean and Human Factors can provide different techniques to improve the code cart’s value and efficiency.

This study sought to improve the code cart’s efficiency, utilization, and cost savings by conducting a holistic review of the cart organization. We hypothesized that reorganizing the code cart with a user-centered design focus would reduce the time needed to find supplies and the cost of the cart.

## METHODS

### Setting

We conducted this study at a quaternary academic pediatric acute care children’s hospital with more than 650 beds, including over 170 combined Neonatal Intensive Care Unit (NICU), Pediatric Intensive Care Unit (PICU), and Cardiac Intensive Care Unit beds. The hospital’s code cart drawers and supplies are standardized throughout the institution except for the NICU’s medication drawer, which differs from the house-wide medication drawers. For this study, we did not change the NICU medication drawer.

### Theoretical Framework

Lean Management and Human Factors were used to help redesign the code cart. Lean Management focuses on improving the value and creating a culture of continuous improvement by reducing waste and increasing the efficiency of a system.^13^ Examples of Lean techniques used include the 5S’s, a system for organizing supplies and establishing flow, where staff walk through a process step by step to determine how to make the task most efficient. Human Factors research studies human performance and its interaction with the environment. Examples of Human Factor techniques used were guided search theory—the study of how humans search for items,^14^ and mistake proofing—the study of how to design items to prevent humans from making a wrong action.^15^ The user-centered design involves the end user at different process steps. Examples of techniques used were: (1) artifact analysis—examining objects involved in a product/process and how users interact; (2) questionnaires/surveys; (3) think-aloud protocol, users talk out loud as they walk through a product/process and discuss pros/cons of it, and (4) usability testing—having users test the new product/process to see what needs to be refined.^16^ Specific usage of each framework can be found in the Results section under Phase 3 Code Cart Redesign.

### Data Collection and Testing

**Fig. 1.**
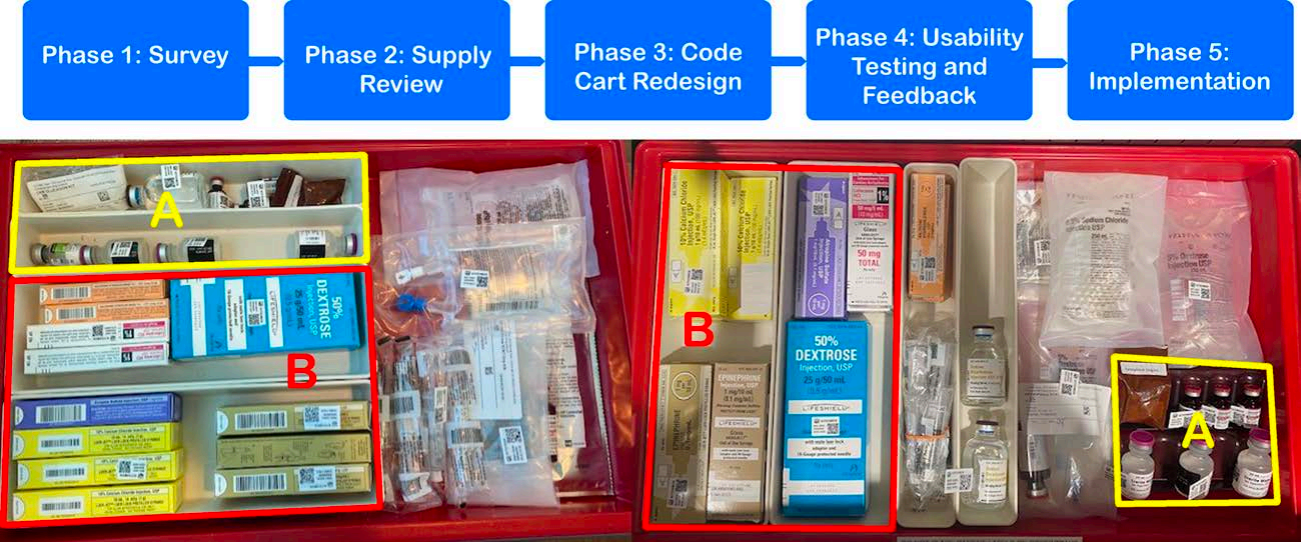
Multiphase approach: example drawer from original code cart on the left and redesigned code cart on the right. A, Mistake-proofed the medication drawer by making it easier to see both the vial names and the names on the boxes. B, Applied search theory and placed the most commonly used medications on the left.

We conducted a multiphase quality improvement project (Fig. 1) using Lean and Human Factors techniques. This study was exempt from IRB as a quality improvement project.

### Phase 1: Staff Survey

We surveyed staff via email who interact with the code cart [eg: PICU/CICU RNs and respiratory therapist (RTs), pharmacy technicians, pharmacists and materials management personnel] (**Figure 1, Supplemental Digital Content** which describes original code cart survey, http://links.lww.com/PQ9/A510.) The survey contained multiple-choice and open-response questions. We analyzed open-response questions by identifying themes.

### Phase 2: Product and Supply Review

Subject matter experts (SMEs) with more than 50 years of accumulative experience in health care, including a pharmacist, a critical care fellow, a material management supervisor, and multiple nurses from the Pediatric Intensive Care Unit and Cardiac Intensive Care Unit, met over 4 hours and reviewed each item in the code cart. This team made lists of necessary, extraneous, and missing supplies using experience from previous codes and structured code reviews. SMEs compared lists until they reached a consensus. Next, the Hospital Code Committee, a multidisciplinary committee encompassing all the disciplines involved in code response and administration, reviewed the supply list to ensure that all the necessary items during a code remained on the code cart.

Finally, supply chain management compared the cost of stocking supplies of the original and new designs. This analysis was performed as a last step to prevent biasing the team from removing more supplies than were necessary.

### Phase 3: Code Cart Redesign

SMEs completed simulated code think-aloud protocol with the original code cart design and discussed staff movements regarding the arrangement and organization of supplies. After removing previously deemed unnecessary supplies, adding needed supplies, and discussing the layout with Human Factors and Lean experts, the team trialed different supply arrangements. We used feedback from the phase one survey, SMEs experience, Human Factors Engineering (mistake proofing and guided search theory), and Lean techniques (establishing flow, eliminating waste, and 5S system) for these trials.^17^

### Phase 4: Usability Testing and Feedback

We conducted basic simulations to test the redesigned code cart to assess how quickly staff could find certain items. We conducted the simulations in the ICU setting to maintain high fidelity. Each room had a bed and a code cart and was set up per the institution’s standard for new admission (ICU rooms contain oxygen hookup, IV pull, suction, etc.). A mannequin was not present for the simulations as it was not needed to test the usability of the original code cart versus the redesigned one.

Each simulation involved 1 RT and 2 ICU RNs that had not seen the redesigned code cart. The PICU Fellow completed advanced simulation training and was the facilitator. The facilitator informed the staff that the objective of the simulation was to test the code cart’s usability by asking them to find certain items and bring them to the patient’s bedside. Before beginning, the facilitator allowed the staff to ask questions and familiarize themselves with the ICU room. Staff were not allowed to look at either of the code carts before the simulation. The facilitator read the following case script at the beginning of every simulation: “There is an 8-year-old patient who is coding, the code alarm has already been pulled, and the team has assembled and started cardiopulmonary resuscitation. The code team leader is asking for 4 things: an appropriate bag and mask, intraosseous (IO) needle supplies, an appropriate dose of epinephrine, and a push/pull fluid bolus set up.” There was no video recording.

**Fig. 2.**
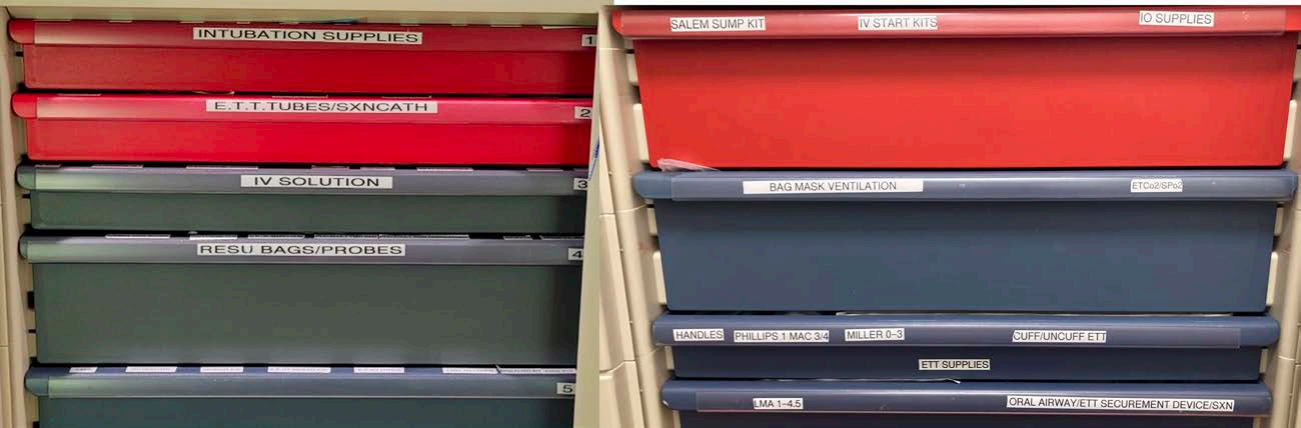
Original vs redesigned code cart: original code cart on the left and redesigned code cart on the right. Previously the drawer colors were randomly chosen. The 5S’s were applied by color coding all the respiratory drawers blue and all the nursing drawers red.

SMEs measured the following metrics: time for RT to find and have the bag valve mask set up to the bed; time for the RN to find and set up the IO and push/pull bolus to the bedside; time for a nurse to find and draw up epinephrine and have it ready for administration (including appropriately labeled); and finally, the overall time to get all items to the bedside. After completing the simulation, we asked staff to complete a survey for feedback regarding the redesigned code cart (**Figure 2, Supplemental Digital Content**, which describes **redesigned code cart survey**, http://links.lww.com/PQ9/A511).

Simulations used the original code cart for the first 5 simulations and the redesigned code cart for the next 8 simulations. Due to provider availability, the last 5 simulations were performed using the existing and redesigned cart, with each trio performing the simulations back-to-back. Half of these simulations first used the original cart design, followed by the redesigned cart. The other half used the redesigned cart first and the original cart design second. For these last 5 simulations, the facilitator randomized the order of the simulations, and the teams were not told which design they would get first. We recruited a convenience sample of voluntary staff members who participated in their normal roles as they would during a real code event. After simulations with the redesigned code cart, the facilitator educated the participants on the redesigned code cart, and they were permitted to look through the drawers and ask questions.

Due to the mixed methods for the time trial, we analyzed the time for each item and the total time using linear mixed models with the Wald test. We analyzed survey results using chi-squared testing for categorical variables and qualitative coding theory for the open-ended questions to identify themes.

### Phase 5: Implementation

Working with all the units, pharmacy, and material management, the SMEs planned a systematic rollout to ensure that each cart had the proper supplies and was consistent across the institution. We provided housewide education regarding the redesigned code carts through housewide education modules and staff huddles. We integrated the new carts into all simulation activities before rollout to gain familiarity. We informed unit managers and charge nurses when their carts would be exchanged and labeled as redesigned code carts to notify staff.

We formed an implementation team of Materials Management staff, SMEs, and volunteers. Each floor must have a working code cart, so Materials Management provided 5 excess code carts to be stocked and reorganized in compliance with the new design and then swapped with original design code carts from the floors. The implementation team then used the retrieved original design code carts to repeat the process throughout the institution. Before each code cart was deployed, Materials Management staff reviewed the counts and expiration dates of the supplies. The pharmacy staff provided the medication drawers for each code cart.

## RESULTS

### Phase 1: Staff Survey

Fifty-seven people (Table 1) participated in the original code cart survey. Phase 4 results include the multiple-choice and scale question analysis.

**Table 1.** Original versus Redesigned Code Cart Survey Results

Job Title			
	Original (%), n = 57	Redesigned (%), n = 37	*P*
Registered nurse	45 (78.9)	26 (70)	0.34
RT	4 (7)	11 (30)	0.003
Pharmacist	0 (0)	0 (0)	0.92
Pharmacy technician	3 (5.3)	NA*	NA
Material management	5 (8.8)	NA*	NA
How often staff grabbed the wrong item or opened the wrong drawer first			
	Original (%), n = 50	Redesigned (%), n = 37	*P*
0–1 times	16 (32)	29 (78)	<0.001
2–3 times	20 (40)	7 (18.9)	
>3 times	14 (28)	1 (2.7)	
Median scale question responses			
	Original	Redesigned	
Items were easily visible:	5	6	<0.001
Could easily find all items needed:	5	6	
Overall code cart was well organized:	5	7	

Using a scale of 1–7 with 1 being strongly disagree and 7 being strongly agree.^2^ Please see **figure 1, Supplemental Digital Content**, http://links.lww.com/PQ9/A510 for full scale.

*Only end users filled out the redesigned survey.

The primary themes of the open responses regarding the original code cart strengths included the availability of supplies at the bedside, the medications being centrally located and in the top drawer, and the general organization of the supplies in the code cart. Weaknesses included not having everything needed, a lack of familiarity with the cart, poor labeling, poor organization, and excess waste either attributed to expiration dates or opening the cart to use only a few items. Suggestions for improvement included removing unnecessary items, better labeling, increasing staff familiarity with the code cart, and adding requested supplies.

### Phase 2: Product and Supply Review

During the second phase, the team reviewed the original code cart design products and focused on the frequency of use, organization, and duplication of items. **Table 1, Supplemental Digital Content**, which describes **Supply Changes to Code Cart**, http://links.lww.com/PQ9/A513 displays the supplies removed or added and the reasons for each. The changes in the code cart amounted to a cost reduction of $878.89 per cart for each full restock. The hospital has 110 active code carts, so the project accumulated aggregated savings of over $96,000.

### Phase 3: Code Cart Redesign

**Fig. 3.**
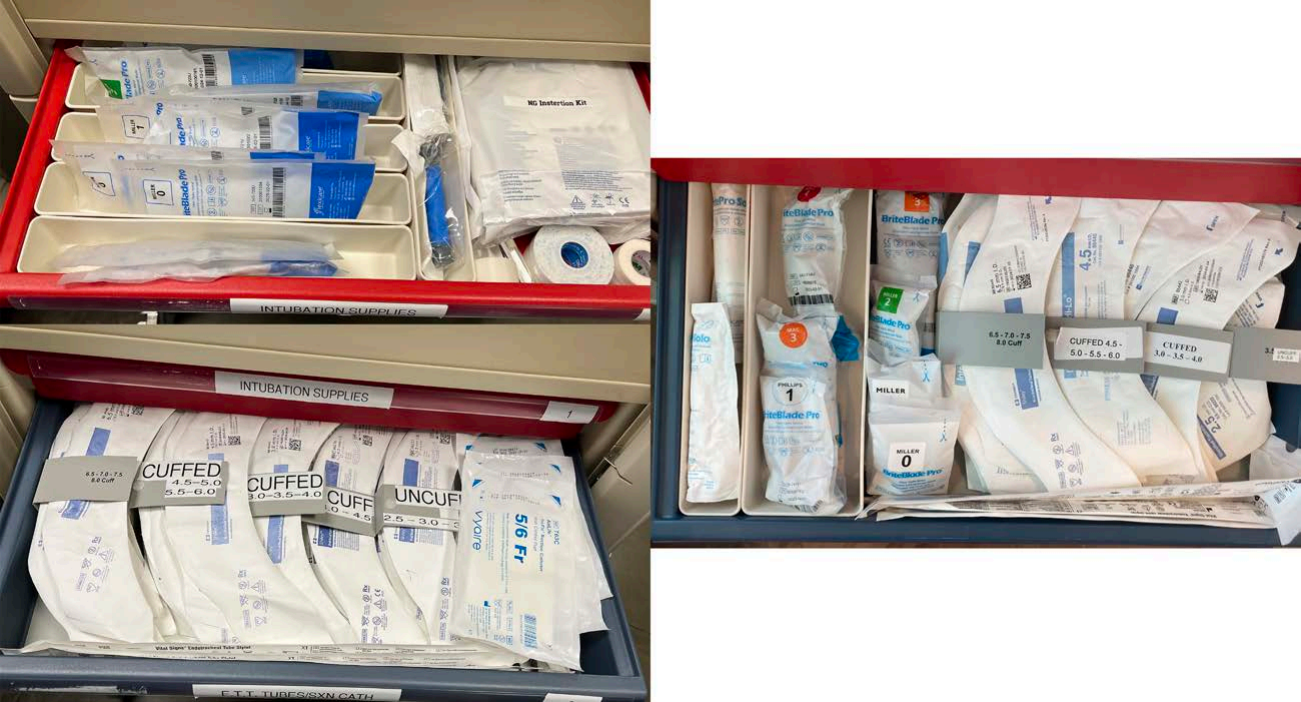
Intubation drawer: original code cart drawers on the left and redesigned code cart on the right. In the original code cart, intubation supplies were spread out over 2 drawers. Used Lean methodology of 5S’s and established flow by placing supplies chronologically for intubation.

**Fig. 4.**
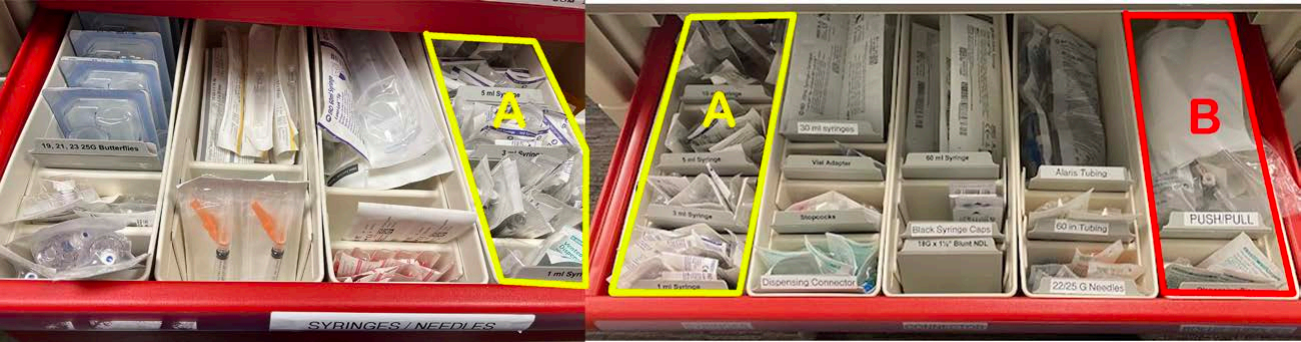
Intravenous supplies: original code cart on the left and redesigned code cart on the right. A, Applied search theory to the nurse supply drawer and placed the most frequently used items on the left. B, Mistake proofed by moving the premade push-pull bolus kit to the nursing supply drawer (instead of in the bottom drawer) so that as nurses obtain supplies for a push-pull, they will see the premade kit.

The team discussed that the original cart design forced staff members to “work over each other” and to look for items in drawers that were on top of each other. The design made items hard to find. In this manner, one staff member’s need to pull certain items would inhibit another staff member from being able to retrieve their needed items. Features of the redesigned code cart used Lean concepts and Human Factors techniques (Figs. 1–4). Examples of how we applied the 5S’s are color-coding RT versus RN drawers and adding more easily readable labels. We established flow by placing all supplies for a task (for example, intubation) in 1 drawer. Guided search theory was applied by placing more frequently used supplies on the left side of each drawer and in relation to one another. Mistake proofing was used by laying medication boxes flat to increase visibility, color-coding medication labels, and adding spice racks for vials so the titles of medications could be read.

### Phase 4: Usability Testing and Feedback

We facilitated 10 simulations with the original cart and 13 with the redesigned cart. The redesigned code cart simulations showed decreased time for each skill and total time (Table 2). However, the only statistically significant result was access to IO equipment (39.1 seconds versus 22.5 seconds, *P* = 0.003).

**Table 2.** Descriptive Statistics Comparing Original and Redesigned Code Cart Design by Item and Total Time

Items Delivered to Bedside	Original Code Cart Times^*^	Redesigned Code Cart Times^*^	*P*
All items (total time)	139.9 ± 41.9	113.8 ± 33.4	0.11
Bag/mask	43.9 ± 31.5	30.1 ± 14.4	0.21
IO equipment	46.36 ± 22.9	23.87 ± 6.6	0.003
Epinephrine	92.17 ± 46.76	95.74 ± 34.63	0.76
IV push/pull system	99.7 ± 46.9	74.43 ± 35	0.15

^*^All times are in seconds.

Survey results showed that staff were less likely to grab the wrong item or open the wrong drawer first in the redesigned code card (*P* < 0.001) (Table 1). In addition, staff thought supplies were more easily visible (5 versus 6, *P* < 0.001), more easily found (5 versus 6, *P* < 0.0001), and overall, the cart was better organized (5 versus 7, *P* < 0.001) (Table 1 and **Figure 3, Supplemental Digital Content,** http://links.lww.com/PQ9/A512).

### Phase 5: Implementation

It took approximately 30 to 45 minutes to rebuild each code cart, and the implementation process was done over 9 weeks.

## DISCUSSION

As health care professionals, it is important to strive to give our patients the best value of care, including high-quality care at a minimal cost. Code carts represent an area where value can be improved. This article highlights the unique collaboration between Lean methodology and Human Factors techniques to redesign the pediatric code cart. This approach combined the efficiency focus of Lean with the cognitive and usability focus of Human Factors, leading to reduced time to obtain lifesaving items, reduced errors in obtaining the correct equipment or opening the correct drawers, and decreased overall cost of the code cart. In addition, not measured in this study is the time it takes Material Management to restock the code carts and how frequently they need to recheck the carts for expired items. The restocking time should be reduced by decreasing the number of items in each code cart. Other institutions can use this phased approach to help improve their code carts and, more importantly, code response.

There are limited publications on the optimal design of code carts. Most facilities organize their pediatric and adult code carts by intervention.^18,19^ One study examined using a color-coded weight-based system to organize resuscitation equipment. They found some improvement in the time it took to obtain intubation equipment and nasogastric tubes.^4^ Other hospitals have used Human Factors techniques to improve the medication drawers showing decreased errors and improved time to obtain medications. Users also found the redesigned medication drawer to be more organized and easier to find medications.^2,14^ A quality improvement initiative in a NICU used Human Factor techniques to revise their code cart and found users preferred the redesigned cart to their previous cart.^18^ After reviewing this literature and discussing it with staff, our institution decided to organize supplies by intervention with a focus on Lean Methodology and Human Factors technique instead of using the color-coded weight-based system. However, further studies could be done on combining these methodologies (Lean, Human Factors, and weight-based color coding).

As health care staff turnover increases, having equipment designed to help the user quickly find needed items without having to rely on experience or memorize the items’ locations can improve time to finding lifesaving measures. Similar to other studies,^14^ the reduction in response time finding supplies did not meet statistical significance but trended toward a statistical result. However, the authors argue that for code events, the clinical significance of this intervention outweighs the statistical significance^19^ as staff reported that they were less likely to grab the wrong item or open the wrong drawer and that the code cart was better organized overall leading to increased usability and better functionality in code events.

This study had limitations. First, we used convenience sampling during the simulation to whatever staff was available on the unit during the simulation. This approach could have contributed to some of our response times not being statistically significant. Second, approximately half of the simulations were conducted back-to-back. It is reasonable to assume that the staff felt more comfortable during the second simulation and possibly acted more quickly during the second simulation because of the refreshed familiarity with the code tasks and skills. We attempted to mitigate this response by randomizing which code cart the team used first during the simulations and using a linear mixed model to analyze the data. Finally, variable levels of experience with the current code cart could have led to bias with how quickly staff members found supplies in the redesigned versus original.^3,16^

Future research should focus on the usability of novice users or using different nonintervention-based code cart designs. In addition, more research is needed to understand the time and cost benefits associated with the restocking of the code cart with a focus on materials management and pharmacy.

## CONCLUSIONS

Patients, employees, and the hospital benefit from the code cart redesign. The redesigned code cart decreased the staff’s time to deliver lifesaving supplies during a simulated code. The code committee will reevaluate the code cart at least every 3 years to examine its usability and if supplies need to be added or removed. This multiphase approach to the redesign was user-driven, provided frontline staff the opportunity to work with and compare the original and redesign code carts, and can be implemented at other institutions. Finally, the cost savings of removing unnecessary supplies will aid in reducing hospital expenditures.

## ACKNOWLEDGMENT

We want to acknowledge all the staff who volunteered their time to help rebuild the redesigned code cart and Bin Zhang for his statistical support.

## DISCLOSURE

The authors have no financial interest to declare in relation to the content of this article.

## Supplementary Material
