# Supplementary material for: Improving Usability of the Pediatric Code Cart by Combining Lean and Human Factors Principles
Source: Pediatr Qual Saf. 2023 Aug 7;8(4):e676. doi: 10.1097/pq9.0000000000000676 (PMC10402944; doi:10.1097/pq9.0000000000000676)
Supplement: Supplementary file 1 [file pqs-8-e676-s001.pdf]

Improving Efficiency and Usability of the Pediatric Code Cart by Combining Lean and Human Factor Principles
First Author: M. Frazier

Figure 2: Original Code Cart Survey

Original Code Cart Survey

1. What is your job title?
    a. RN
    b. RT
    c. Pharmacy Tech
    d. Material Management
    e. Pharmacist
    f. Emergency Tech

2. How long have you been working with the code cart?
    a. <1 year
    b. 1-3 years
    c. 3-5 years
    d. >5 years

3. What do you think the strengths of the current code cart are?

4. What do you think the weaknesses of the current code cart are?

5. Do you have any suggestions to improve the code cart?

6. If you are clinical, approximately how many times when using the code cart do you find yourself having to open multiple drawers or initially grabbing the wrong item before finding what you were looking for? For example: I'm looking for sodium bicarb but grab calcium first. Or I'm trying to find the IO but look in drawer 4 instead of drawer 5.
    a. 0-1 times
    b. 2-3 times
    c. >3 times

7. If you are clinical, using the following scale how much do agree with the following comments:
    a. The items I needed were easily visible
    b. I could easily find all the items I needed
    c. Overall, the code cart was well organized

Strongly Disagree	Disagree	Slightly Disagree	Neutral	Slightly Agree	Agree	Strongly Agree
1	2	3	4	5	6	7
