# Supplementary material for: Improving Usability of the Pediatric Code Cart by Combining Lean and Human Factors Principles
Source: Pediatr Qual Saf. 2023 Aug 7;8(4):e676. doi: 10.1097/pq9.0000000000000676 (PMC10402944; doi:10.1097/pq9.0000000000000676)
Supplement: Supplementary file 2 [file pqs-8-e676-s002.pdf]

Improving Efficiency and Usability of the Pediatric Code Cart by Combining Lean and Human Factor Principles
First Author: M. Frazier

Supplemental Figure 2: Redesigned Code Cart Survey

Redesigned Code Cart Survey

1. What is your job title?
   a. RN
   b. RT
   c. Pharmacist
   d. Emergency Technician
2. Approximately how many times when using the code cart did you find yourself having to open multiple drawers or initially grabbing the wrong item before finding what you were looking for? For example: I'm looking for na bicarb but grab calcium first. Or I'm trying to find the IO but look in drawer 3 instead of drawer 4.
   a. 0-1 times
   b. 2-3 times
   c. >3 times
3. Using the following scale how much do agree with the following comments:
   a. The items I needed were easily visible
   b. I could easily find all the items I needed
   c. Overall, the code cart was well organized

Strongly Disagree	Disagree	Slightly Disagree	Neutral	Slightly Agree	Agree	Strongly Agree
1	2	3	4	5	6	7

4. Any other feedback on the redesigned code cart (good or bad)?
