# Supplementary material for: Improving Usability of the Pediatric Code Cart by Combining Lean and Human Factors Principles
Source: Pediatr Qual Saf. 2023 Aug 7;8(4):e676. doi: 10.1097/pq9.0000000000000676 (PMC10402944; doi:10.1097/pq9.0000000000000676)
Supplement: Supplementary file 3 [file pqs-8-e676-s003.pdf]

Improving Efficiency and Usability of the Pediatric Code Cart by Combining Lean and Human Factor Principles

First Author: M. Frazier

Supplementary Table 1: Supply Changes to Code Cart

Drawer	Items removed	Reason
1	Duplicates of intubation blades	Duplicate blades unnecessary
	Large tegaderm kit in NG kit	Replaced with a smaller size tegaderm in NG kit
	Sterile Gloves in NG kit	Sterile gloves are not needed for intubation or to place a nasogastric tube. If sterile gloves are needed for another procedure, they can be gathered from another area with other needed supplies.
	Tape	Removed par from 5 to 3 (removed larger thicknesses)
	Yankeur suction	Reducing par from 2 to 1
2	Larger uncuffed ETT	3.5, 4.0, and 4.5 uncuffed ETT are not used in clinical practice frequently. A downsized cuff ETT would be preferred if ETT was too big.
3	500 ml normal saline bag	500 ml normal saline. This was a redundant item. A 1L Normal saline bag was left.
	5 in and 10 in IV tubing extension	5 in extensions tubing was redundant as it is included in the IV starter kits that are on the code cart<br>10 in extensions are rarely used.
	Scalpel	There is no procedure that can be performed with the code cart alone that needs a scalpel. If a procedure that requires a scalpel is being performed, supplies will be acquired from another area.
	Arm boards	Arm boards aren’t used during codes, usually placed after the code is resolved
4	Pediatric and Adult nonrebreather masks	In a code, bag/mask ventilation is used. Nonrebreather masks should not be used.
5	Gloves	Gloves are located in and outside of every patient room

	Surgical Masks	Surgical masks are located in and outside of every patient room
	1 (23 gauge) inch and 1.25 inch (22 gauge) arterial blood gas needles	We do not use these for ABG anymore. We would use an IV start needle in an acute situation
	Emesis bags	In a code, suction is usually used when a patient vomits
**6 (medication drawer)**	100ml normal saline bag	Replaced with 250 ml normal saline bag
	Glucagon	Code cart contains d25 or d50 to be used instead
	Vecuronium	Rocuronium is already present
**7**	Device access multidose needle free	Reduced par from 10 to 4 as 10 were not needed for a code response
	1 in needles for IM epinephrin	Reduced par from 10 to 3 as 10 were not needed for a code response
	Butterfly needles	Staff use IV start needles in codes
	Vacutainers	Staff draw blood in syringes for labs during codes
	Insulin syringes	Meds are drawn with 1- or 3-ml syringes
	Blunt tip needles	Reduced par from 25 to 15
	50 ml catheter tip needle	Use 60 ml luer lock syringes instead
	10 ml syringes	Reduced par from 15 to 10
	Finger stick lancets	Replaced with heal stick lancets
	**Items Added**	**Reason**
3	16-24 guage IV start needles	Increased par from 1 to 2 in both infant and pediatric IV start kit. To consolidate supplies and removed from cart to decrease redundancy elsewhere
7	1 ml and 3 ml luer lock syringes	Increased par from 15 to 20 as they ear frequently used
	30 ml luer lock syringes	Increased par from 4 to 10
	Luer lock syringe caps	Increased from 30 to 50 as they are frequently used
**Supplies Not currently**	8 french replogle	Added this size replogle for our smallest patients

located on cart		
	14 gauge needle	Added for needle decompression of pneumothorax
	Heal stick lancets	Removed finger stick lancets and added heal stick lancets that puncture deeper.
	25 gauge 1.5 inch safety needle	For administration of IM epinephrine
	Dispensing connectors	Help connect bristojets directly to syringes
